# Initial Evaluation of Computer-Assisted Radiologic Assessment for Renal Mass Edge Detection as an Indication of Tumor Roughness to Predict Renal Cancer Subtypes

**DOI:** 10.1155/2019/3590623

**Published:** 2019-04-23

**Authors:** Rahul Rajendran, Kevan Iffrig, Deepak K Pruthi, Allison Wheeler, Brian Neuman, Dharam Kaushik, Ahmed M Mansour, Karen Panetta, Sos Agaian, Michael A. Liss

**Affiliations:** ^1^University of Texas San Antonio, Department of Electrical and Computer Engineering, San Antonio, TX, USA; ^2^University of Texas Health San Antonio, Department of Urology, San Antonio, TX, USA; ^3^Tufts University, School of Engineering, Medford, MA, USA; ^4^College of Staten Island, The City University of New York (CSI-CUNY), New York City, NY, USA; ^5^University of Texas San Antonio, Department of Biology, San Antonio, TX, USA

## Abstract

**Objective:**

To develop software to assess the potential aggressiveness of an incidentally detected renal mass using images.

**Methods:**

Thirty randomly selected patients who underwent nephrectomy for renal cell carcinoma (RCC) had their images independently reviewed by engineers. Tumor “Roughness” was based on image algorithm of tumor topographic features visualized on computed tomography (CT) scans. Univariant and multivariant statistical analyses are utilized for analysis.

**Results:**

We investigated 30 subjects that underwent partial or radical nephrectomy. After excluding poor image-rendered images, 27 patients remained (benign cyst = 1, oncocytoma = 2, clear cell RCC = 15, papillary RCC = 7, and chromophobe RCC = 2). The mean roughness score for each mass is 1.18, 1.16, 1.27, 1.52, and 1.56 units, respectively (*p* < 0.004). Renal masses were correlated with tumor roughness (Pearson's, *p*=0.02). However, tumor size itself was larger in benign tumors (*p*=0.1). Linear regression analysis noted that the roughness score is the most influential on the model with all other demographics being equal including tumor size (*p*=0.003).

**Conclusion:**

Using basic CT imaging software, tumor topography (“roughness”) can be quantified and correlated with histologies such as RCC subtype and could lead to determining aggressiveness of small renal masses.

## 1. Introduction

Kidney cancer (renal cell carcinoma; RCC) is expected to account for 3.8% of all new cancers diagnosed in 2017 and will be responsible for an estimated 14,400 deaths [1]. The widespread use of radiologic imaging likely accounts for the increased incidence of new cancer cases diagnosed in the United States since the 1970s. Up to 40% of these incidental masses are localized, and approximately 15–20% of these small renal masses are ultimately benign. [2–7].

Small renal masses are typically not aggressive and are slow growing with low malignant potential; therefore, urologists have adapted a monitoring program to follow these masses over time called active surveillance [8]. Active surveillance heavily relies on growth of tumors in size and lacks uniform standardization usually citing every 6-month imaging [9]. While size is an important factor in renal tumors, it does not solely predict the malignant potential of a small renal mass. In a study by Daugherty et al., proposed the need for different size cutoffs for the various renal cancer subtypes indicating the clear cell cancer has the most malignant potential and should be smaller than 4 cm and chromophobe has the lowest malignant potential and could wait until 7 cm size [10]. While active surveillance in appropriately selected patients is a viable alternative to surgical intervention, it still carries the burden of uncertainty, patient anxiety, risk of disease progression, and the associated financial cost to the patient [11]. As the diagnosis of small renal masses remains primarily radiographic, the development of software to assess the potential aggressiveness of a mass would be a useful aid in the clinicians' armamentarium for shared decision-making. Previous studies have attempted to discern tumor histology through the use of current imaging techniques and automated image analysis with varying degrees of success [12–16].

In order to provide additional information on malignant potential of small renal masses without needing a biopsy, we utilize computed tomographic (CT) scans with image enhancement techniques and edge detection to calculate the surface irregularities of renal masses scheduled for partial nephrectomy. Using this technology, we create an overall “Tumor Roughness Score” (TRS) to determine the distinction of renal tumor subtype and potential aggressiveness. The initial assessment of this technique is to determine its utility in active surveillance clinical follow-up and potential research trials.

## 2. Methods

### 2.1. Patient Population

Thirty patients who underwent nephrectomy for RCC from our institution represent cases of clear cell RCC (*n*=10), papillary RCC (*n*=10), and chromophobe RCC (*n*=10). Limited demographic and follow-up data were available. We excluded patients with known hereditary disorders and those deemed to have poor quality CT images.

### 2.2. Tumor Roughness Measure by Using Multilevel Voxel Box Counting

The “roughness” measure of a kidney tumor requires contrast-enhanced CT scans, which automatically detect, quantitatively measure, and distinguish lymph node involvement and local tumor invasion based on the tumor's topography [17]. The extensive computer simulation validates the proposed roughness measure acquisition that requires a three-stage algorithm.

#### 2.2.1. Improving the Image Quality and Contrast

In general, the quality of images is affected by factors such as restricted image dimension and pixel resolution along with the compulsive effects caused due to noise and poor contrast. To overcome these problems, we utilize well-described enhancement methods such as guided filtering, edge enhancement, and image fusion to improve the contrast and quality of the image [18, 19].

#### 2.2.2. Enhanced Image Multilevel Segmentation Using Its Alpha-Trimmed Mean and Variance

Multiple-level segmentation aims to obtain more than one threshold for a given image and segment the image into specific regions of interest (tumor) overlying one background [20]. The method assumes that the image contains only foreground and background information. Traditional multilevel segmentation techniques are usually susceptible to Gaussian and impulse noise. The algorithm is a modification of the multilevel thresholding technique for image segmentation that eliminates noise and performs a comprehensive search for a threshold that minimizes the intraclass (foreground and background) alpha-trimmed variance [21]. The critical component of this method is the selection of a threshold such that it separates the pixels into multiple classes by maximizing the between-class variance. The between-class variance is defined as

(1) $$\begin{array}{c} \sigma^{2}=\;\;\overset{M}{\underset{i=1}{\sum }}P_{i}\left(\mu_{i}-\mu_{\text{T}}\right), \end{array}$$

where *σ*^2^ is the between-class variance, *P*_*i*_ is the probability of class *i*, *μ*_*i*_ is the mean value of class *i*, and *μ*_T_ is the total mean of the histogram.

#### 2.2.3. Computation of the Average of Hausdorff's Measure of the Regions of Interest (Tumor)

The Hausdorff measure is a scale of closeness of two groups of points that are subclasses of a metric space [22]. The measure assigns a scalar score to the similarity between two paths, data clouds, or any group of points. It is defined by equations (2) and (3). Let (*A*, *d*) be a metric space. For any subset *M* ⊂ *A*, diam(*M*) will denote the diameter of *M*. For any *M* ⊂ *A*, any *δ* ∈ [0, *∞*], and any *α* ∈ [0, *∞*], the outer measure is given by

(2) $$\begin{array}{c} H_{\delta }^{\alpha }\left(M\right)=\inf\left\{\overset{\infty }{\underset{i=1}{\sum }}\text{diam}{\left(M_{i}\right)}^{\alpha }:M\subset \underset{i}{\cup }E_{i}\;\;\mathrm{and diam}\left(M_{i}\right)<\delta \right\}. \end{array}$$

Hence, the Hausdorff *α*-dimensional measure of *M* is

(3) $$\begin{array}{c} H^{\alpha }\left(M\right)=\;\;\underset{\delta ⟶0}{\lim}H_{\delta }^{\alpha }\left(M\right). \end{array}$$

The system uses the box counting method to generate feature points based on the presence of edges in the image. Based on these feature points, the Hausdorff dimension is estimated and the slope of the best fit line generates a roughness score. In our case, Hausdorff's measure is an outer measure that calculates the roughness of each level and a combined score is generated. This score is referred to as the Tumor Roughness Score.

## 3. Statistical Analysis

Continuous variables were analyzed with either *T*-tests or ANOVA for comparisons of the five renal mass subtype groups. ANOVA post hoc analysis used least significant difference (LSD). All *p* values are two-tailed with a significance of *p* < 0.05, unless otherwise specified. The Pearson correlation coefficient is used to determine correlation analysis specifically between renal mass size and tumor roughness because larger tumors are more likely to be aggressive. We utilized a linear regression model to identify the factors that are most influential on predicting tumor subtype.

## 4. Results

**Figure 1:**
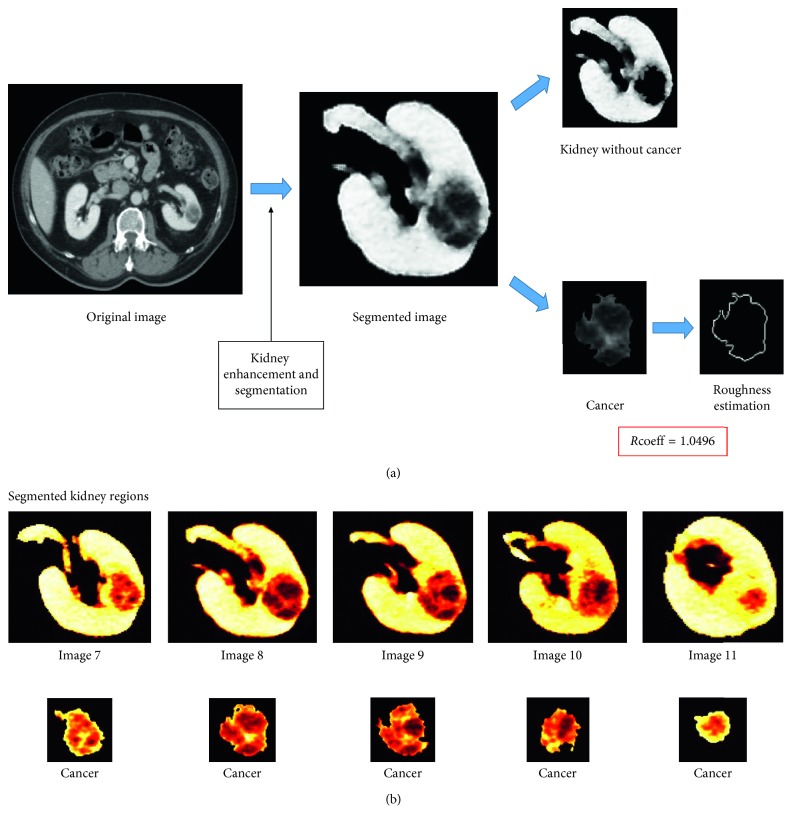
Tumor roughness score image analysis. Adaptive segmentation technique developed to segment the kidney and tumor for surface enhancement and investigate tumor irregularity. The summary irregularity score (*R*_avg_ = 1.0413) is averaged across all segments of the tumor.

Of the thirty patients selected, we excluded three patients due to poor quality CT images. We selected the data from 27 patients for analysis composed of the following subtypes: benign cyst (*n*=1), oncocytoma (*n*=2), clear cell RCC (*n*=15), papillary RCC (*n*=7), and chromophobe RCC (*n*=2). Overall demographics include median age of 56 (IQR 48–63), median body mass index of 30.4 (27.6–32.0), median tumor size of 2.5 cm (IQR 1.8–4 cm), and four positive margins (14.8%, 4/27) and consisted of 93% men (25/27). Only one tumor was upgraded to pT3a and a total of three to pT1b, with all other tumors being pT1a. Race/ethnicity includes 59% of European descent and 41% of non-European descent. We display the demographics of each subtype and comparisons in Table 1. At a median follow-up of 3.9 years (interquartile range (IQR) 2.2, 5.4 years), there were no patient deaths. None of the patients had a documented recurrence. The majority of the cases were stage 1 disease; two cases did not have a recorded stage, and none of the subjects had documented metastasis or received neoadjuvant therapy before surgical resection (Table 1). We display an example of the adaptive segmentation technique for image processing for the calculation of the tumor roughness score in Figure 1. We obtain standard clinical CT scans and segment the kidney and tumor for surface enhancement separately. To investigate tumor irregularity, we take the tumor slice by slice using edge detection and other techniques described in Methods to obtain a 3-dimensional surface irregularity. These slices are calculated into a composite score of tumor roughness.

**Figure 2:**
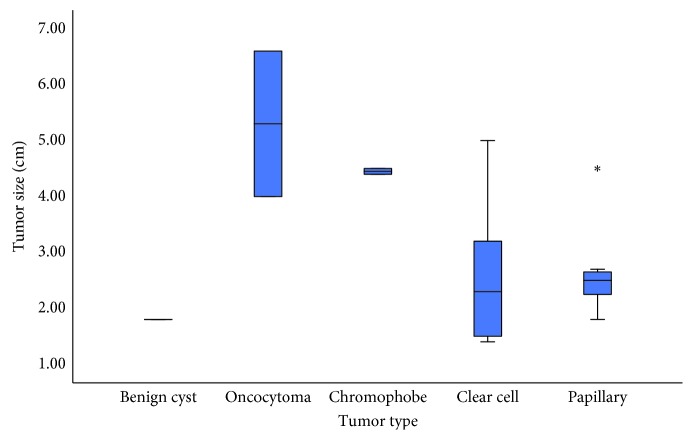
Box and whisker plot of renal mass size compared to the renal tumor subtype for trend.

**Figure 3:**
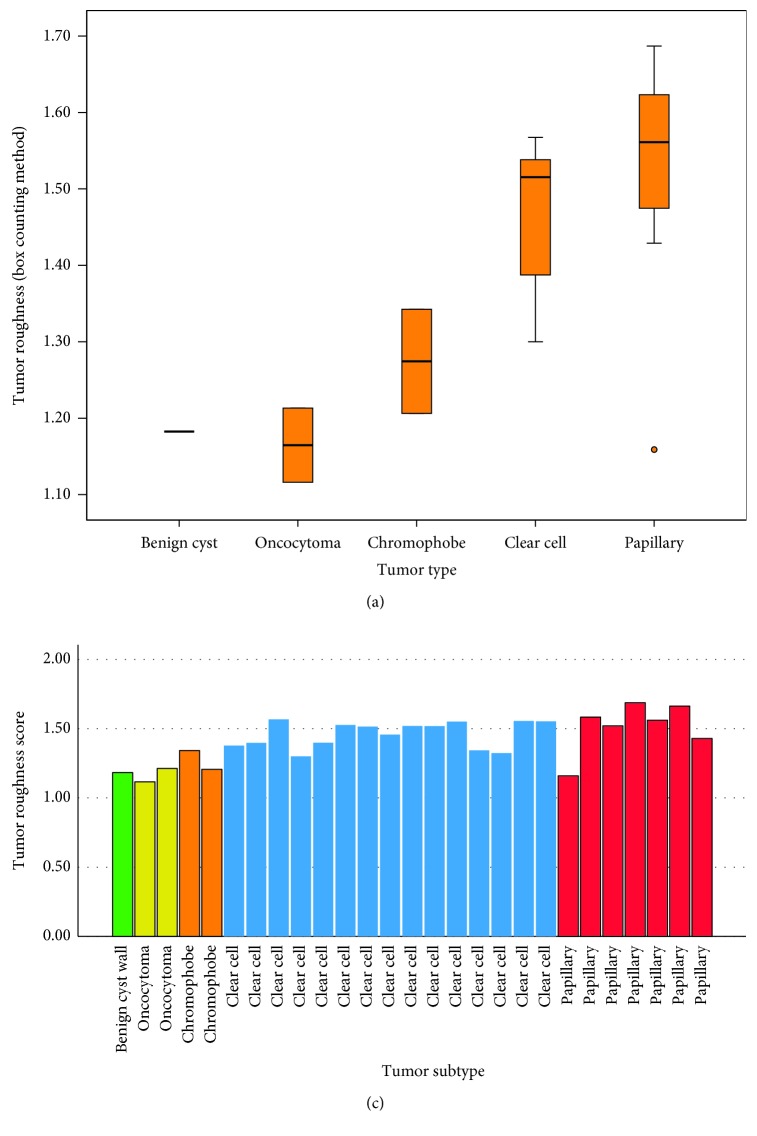
(a) Box and whisker plot displaying the tumor surface roughness score compared to each renal mass subtype for trend. (b) Waterfall plot for tumor roughness score with each renal tumor subtype listing less aggressive tumors to the left and more aggressive tumors to the right.

**Figure 4:**
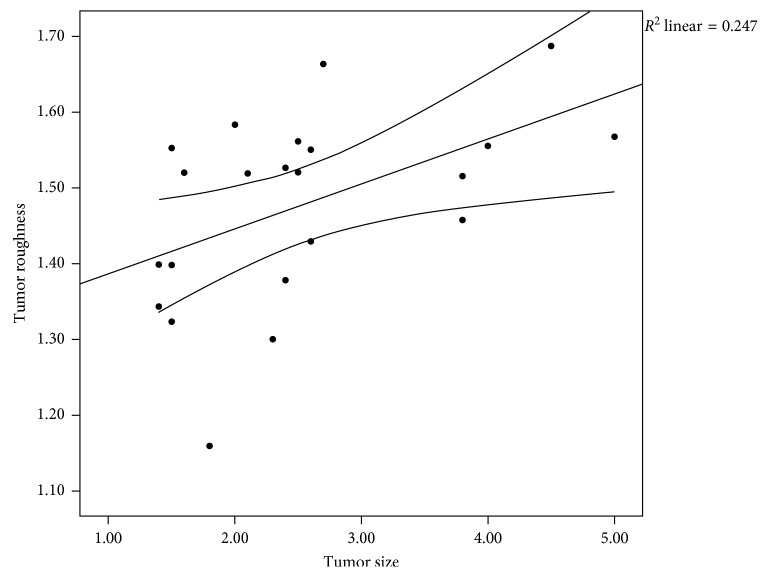
Scatter plot noting the correlation between renal mass size and tumor roughness score (Pearson's *p*=0.02).

Of the 27 patients, tumor size associated with benign renal masses (Figure 2, *p*=0.01). The mean roughness score for each mass is 1.18, 1.16, 1.27, 1.52, and 1.56 units, respectively (Figure 3(a), *p* < 0.004). The mean TRS did trend along the renal cancer subtype associated with tumor aggressiveness. The lowest quartile is 1.32 and would indicate the lowest risk based on this dataset and is visually displayed in the waterfall plot of TRS and colored by the corresponding tumor subtype. The stage T3a-upgraded tumor has a TRS of 1.46. The mean scores of positive and negative margins were identical (*p*=0.9), but both above the lowest quartile. We investigated those tumors with Furman score (*n*=23) and did not show significant differences between high grade tumors vs. low grade tumors (*p*=0.7); however, 83% (19/23) were documented as Furman Grade 2 (low grade) and therefore, there was not an acceptable range of tumor cellular aggressiveness based on histopathology. Renal masses size correlates with tumor roughness (Pearson's, *p*=0.02, Figure 4). However, tumor size itself was larger in benign tumors rather than malignant tumors (*p*=0.01) giving the indication that size would not be a significant factor for aggressiveness. Linear regression analysis noted that the roughness score is the most influential on the model with all other demographics being equal including tumor size (*p*=0.003) (Table 2).

## 5. Discussion

Herein, we describe a data image processing technique to provide a tumor roughness score that can have additional information beyond tumor size. Currently, physicians use tumor size and growth rate in active monitoring strategies. However, this strategy has not shown to be accurate regarding tumor subtype or tumor aggressiveness. We show that the TRS can be a useful adjunct to image assessment and has the potential for automation using artificial intelligence and machine learning. TRS provides additional information regarding tumor subtype which correlates with the traditional RCC aggressiveness without a renal mass biopsy. This information is already available to physicians; however, computer-assisted techniques remain limited for evaluation. The roughness scoring could provide information regarding those tumors that may be less aggressive from the clear cell and papillary cancers. We plan on using these techniques in future clinical trials and to potentially implement machine learning protocols, which would enhance its predictive ability with more comprehensive datasets.

We distinguish our analysis from previous manuscripts describing the surface contact area, which has also been shown to predict postoperative outcomes but was not investigated regarding tumor subtype [23, 24]. Tumor surface contact areas only represent how much tumor is touching the kidney parenchyma, and essentially, the test is an indicator of how exophytic a renal tumor is to its surrounding tissue. Our technique enhances and outlines the entire tumor surface for irregularity not based on exophytic or endophytic which is usually reserved for surgical resection planning. Another utilization of CT imaging is the texture analysis performed by Khene and colleagues noting the texture on CT could predict malignant or benign tumors as well as adherent perinephric fat during surgical intervention [25, 26]. Closure to our analysis is the work performed by Linguraru et al. who developed semiautomated quantification and classification of renal tumors to classify benign or malignant tumors especially those patients with genetic abnormalities prone for forming multiple kidney tumors [27]. Considerable effort has been placed to detect papillary renal cancer from clear cell including subtypes based on contrast enhancement largely because papillary renal neoplasms have a poor or delayed enhancement that can be difficult to discern from hyperdense renal cysts [28, 29]. In addition, papillary subtype tumors are less likely to have a thick pseudocapsule around the tumor which would coincide with having more irregular edges to the tumor [30]. Our technique could assist with this particular question in more extensive studies.

Data science with the use of deep learning based-automated methods is ever increasing and merging with clinical care [31]. Other authors have utilized similar techniques for image analysis. Pattern recognition is essential in radiology; however, utilization of quantitative image analysis will provide objective additional data on tumor aggressiveness. These techniques have been applied in lung cancer and others [32–34]. A review on other techniques for small renal mass characterization will be utilized in further evaluation and improvements [35, 36]. We seek to add to the expanding literature using image analysis techniques to utilize data science to implement actionable data to physicians and patients.

There are some limitations to this study. First, while we identified some potentially significant associations, this is a pilot study in a small cohort of patients without validated findings. Secondly, our cohort included CT scans performed at different institutions using different CT scanners, which could insert variability into the quality of the roughness score. However, in practical use, this would be the norm as other clinicians would utilize CT images from their home institutions. We assume that the tumor roughness score will have more applicability in small renal masses to have more influence on future decision making. Unfortunately, we are unable to compare our method with methods of previous authors because specific algorithms have not been published [37]. We acknowledge the fast moving field of image analysis and new techniques to separate target lesions in imaging with new techniques such as topographical assessments [38]. We included all renal mass types because until surgical removal, the subtype is completely unknown, giving us impetus for this study. We are currently developing validation profiles of our work and development of AI technology that can provide visual tumor characteristics in quantitative form as a biomarker.

## 6. Conclusion

Using existing CT imaging in this proof of concept study, renal mass tumoral topography can be quantified and correlated with histology and biological aggressiveness in small renal masses. We provide preliminary data to suggest the use of imaging topography could provide additional information in the selection of patients for active surveillance or monitoring of renal masses compared to more aggressive therapy. A larger study is warranted to validate these findings and to determine if radiographic tumor surface analysis can obviate the need for conventional serum or urine biomarkers.

## Figures and Tables

**Table 1:** Demographics by tumor subtype.

Renal cancer subtype	Benign cyst	Oncocytoma	Chromophobe	Clear cell	Papillary	*p* value
Number of patients (*N*)	1	2	2	15	7	
Age	60	45 (42–45)	54 (51–54)	56 (42–66)	57 (51–63)	0.9
Body mass index	24.9	26.3 (24.1–26.3)	31.8 (31.3–31.8)	30.1 (27.3–31.6)	32.01 (29.9–40.7)	0.05
Race/ethnicity (white %)	1/1 (100%)	1/2 (50%)	0/2 (0%)	9/15 (60%)	5/7 (71%)	0.4
Biologic gender (male %)	0/1 (0%)	1/2 (50%)	2/2(100%)	15/15 (100%)	7/7 (100%)	0.001
Tumor size (cm)	1.8	5.3 (4.0–5.3)	4.45 (4.4–4.45)	2.3 (1.5–3.8)	2.5 (2.0–2.7)	0.01
Tumor stage (stage 3)	0/1 (0%)	0/2 (0%)	0/2 (0%)	1/15 (7%)	0/7 (0%)	0.3
Tumor surface roughness	1.18	1.16 (1.12–1.16)	1.27 (1.21–1.27)	1.52 (1.38–1.55)	1.56 (1.43–1.66)	0.004

**Table 2:** Multivariable linear regression model or the outcome of kidney cancer subtype.

Demographic	Coefficient (95% CI)	*t* score	*p* value
Constant	2.59 (−5.8–0.58)	−1.709	0.1
Age	0.004 (0.17–0.025)	0.9	0.69
Body mass index	0.049 (0.008–0.107)	2.7	0.09
White (yes vs. no)	0.114 (0.364–0.592)	0.5	0.62
Tumor size	1.71 (−0.351–0.009)	−1.2	0.06
Roughness score	2.601 (0.977–4.225)	4.2	0.003

## Data Availability

The data used to support the findings of this study are available from the corresponding author upon request.
